# Protocol: identifying policy, system, and environment change interventions to enhance availability of blood for transfusion in Kenya, a mixed-methods study

**DOI:** 10.1186/s12913-023-09936-0

**Published:** 2023-09-07

**Authors:** Alejandro Munoz-Valencia, Jackline O. Aridi, Linda S. Barnes, Kristina E. Rudd, Bopaya Bidanda, Tonny Epuu, Robert Kamu, Tecla Kivuli, Jana Macleod, Cindy M. Makanga, Jennifer Makin, Muthoni Mate, Carolyne Njoki Muiru, Gatwiri Murithi, Abdirahaman Musa, Hellen Nyagol, Kevin Ochieng, Jayant Rajgopal, Nakul P. Raykar, Yiqi Tian, Mark H. Yazer, Bo Zeng, Bernard Olayo, Pratap Kumar, Juan Carlos Puyana

**Affiliations:** 1 Department of Anesthesiology and Perioperative Medicine, University of Pittsburgh, Pittsburgh, PA USA; 2 Institute of Healthcare Management, Strathmore University Business School, Nairobi, Kenya; 3 Linda S. Barnes Consulting, Seattle, WA USA; 4 Doctor of Public Health Leadership, University of Illinois-Chicago, Chicago, IL USA; 5 Clinical Research, Investigation, and Systems Modeling of Acute Illness (CRISMA) Center, Department of Critical Care Medicine, University of Pittsburgh, Pittsburgh, PA USA; 6 Department of Industrial Engineering, University of Pittsburgh, Pittsburgh, PA USA; 7 Department of Surgery, Kenyatta University, Nairobi, Kenya; 8 Department of Obstetrics, Gynecology, and Reproductive Sciences, University of Pittsburgh Medical Center Magee Women’s Hospital, Pittsburgh, PA USA; 9 Center for Public Health and Development, Kisumu, Kenya; 10 Department of Surgery, Egerton University, Nakuru, Kenya; 11 Ministry of Health & Sanitation, Turkana County Government, Turkana, Kenya; 12 Department of Surgery, Brigham and Women’s Hospital, Boston, MA USA; 13 Program in Global Surgery and Social Change, Harvard Medical School, Boston, MA USA; 14 Center for Surgery and Public Health, Brigham and Women’s Hospital, Boston, MA USA; 15 Department of Pathology, University of Pittsburgh, Pittsburgh, PA USA; 16 Departments of Surgery and Critical Care Medicine, University of Pittsburgh, Pittsburgh, PA USA

**Keywords:** Blood transfusion, Blood supply, Bleeding, Anemia, Process mapping, Industrial engineering, Policy system and environment change, Mixed-methods, Kenya, Sub-Saharan Africa

## Abstract

**Background:**

Safe blood is essential for the care of patients with life-threatening anemia and hemorrhage. Low blood donation rates, inefficient testing procedures, and other supply chain disruptions in blood administration affect patients in low-resource settings across Sub-Saharan countries, including Kenya. Most efforts to improve access to transfusion have been unidimensional, usually focusing on only point along the blood system continuum, and have excluded community stakeholders from early stages of intervention development. Context-appropriate interventions to improve the availability of safe blood at the point of use in low-resource settings are of paramount importance. Thus, this protocol proposes a multifaceted approach to characterize the Kenyan blood supply chain through quantitative and qualitative analyses as well as an industrial engineering approach.

**Methods:**

This study will use a mixed-methods approach in addition to engineering process mapping, modeling and simulation of blood availability in Kenya. It will be guided by a multidimensional three-by-three-by-three matrix: three socioeconomic settings, three components of the blood system continuum, and three levels of urgency of blood transfusion. Qualitative data collection includes one-on-one interviews and focus group discussions with stakeholders across the continuum to characterize ground-level deficits and potential policy, systems, and environment (PSE) interventions. Prospectively-collected quantitative data will be used to estimate blood collection and transfusion of blood. We will create a process map of the blood system continuum to model the response to PSE changes proposed by stakeholders. Lastly, we will identify those PSE changes that may have the greatest impact on blood transfusion availability, accounting for differences across socioeconomic settings and levels of urgency.

**Discussion:**

Identifying and prioritizing community-driven interventions to improve blood supply in low-resource settings are of utmost importance. Varied constraints in blood collection, processing, delivery, and use make each socioeconomic setting unique. Using a multifaceted approach to understand the Kenyan blood supply and model the response to stakeholder-proposed PSE changes may lead to identification of contextually appropriate intervention targets to meet the transfusion needs of the population.

**Supplementary Information:**

The online version contains supplementary material available at 10.1186/s12913-023-09936-0.

## Background

Blood for transfusion, which includes whole blood as well as blood components (blood products), has been recognized as an essential medicine by the World Health Organization (WHO) since 2013, and its availability is vital to meet the priority health needs of a population [1]. To achieve a sufficient supply of blood products available for timely and safe blood transfusion, public health experts advance blood donation from voluntary non-remunerated blood donors (VNRBD) [2]. Despite the global need, there is wide country- and region-level variation in blood collection, with low- and middle-income countries (48% of the global population) collecting only 24% of the global donations and high-income countries (19% of the global population) collecting 47% of global donations [3]. The 2016 WHO Global Status Report on blood safety and availability reported that just 5.6 million units of blood, 5% of the current global supply, were collected in Africa, which represents 13% of the global population [3]. Blood collection rates in Sub-Saharan Africa are ten-fold lower than rates in high-income countries, and as of 2013, 38 of 47 African countries collected less than the WHO recommended target of 10 whole blood donations per 1,000 population per year [3]. Moreover, of the blood that is collected, most exists in urban blood banks, leaving little for rural populations [4]. In low and lower-middle income countries (LLMICs) hospitals, the unavailability of banked blood hampers timely blood transfusion, meaning that most facilities resort to family replacement donation (FRD), a subsistence approach relying on blood donation from family members, to support individual patient needs [5].

From 2004 through 2019 the United States President’s Emergency Plan for AIDS Relief (PEPFAR) program provided funding to develop and strengthen national blood banking systems in 14 sub-Saharan African countries, including Kenya [6]. This ultimately led to the technical assistance and financial support for the Kenya National Blood Transfusion Service (KNBTS), a decentralized but coordinated blood banking system, created in 2000 [7]. The KNBTS works under the Ministry of Health and is mandated to provide safe and adequate blood products in the country. The KNBTS structure consists of a national coordinating unit; six Regional Blood Transfusion Centers (RBTCs) that conduct blood collection, testing, storage, and distribution; and 11 satellite centers that only collect blood and rely on RBTCs for testing. Furthermore, management of blood practices and products at the county level are also coordinated with County Health Management Teams (CHMTs). Kenya has an annual blood need of approximately 530,000 units based on the WHO formula of 1% of the country’s total population, currently estimated at 53 million [8]. According to KNBTS, collections reached just 30% of this mark as of 2017 [9]. In addition to the availability deficit, inefficiencies in supply chains result in shortages of reagents for testing and wastage of collected blood [10]. Even for patients for whom blood transfusion is available, delays are common [11]. The consequences of such shortfalls include excessive morbidity and mortality of patients with bleeding conditions such as postpartum hemorrhage and injuries as well as anemia from malaria and sickle cell disease. Therefore, optimizing the blood system continuum (blood collection, processing, delivery, and use) in Kenya represents both a major health challenge and a social imperative [12].

Previous efforts to address blood availability worldwide focused on increasing VNRBD, testing for infectious agents transmitted through transfusion blood processing, enhancing blood delivery, and optimizing clinical blood use [12]. Examples include iron supplementation [13], mobile blood donation drives [14], blood donation incentives [15], improved testing for transfusion transmissible infections (TTI) [16], componentization of blood units [17], standardization of blood banking procedures [18], policies promoting donation and quality improvement [19], formation of hospital blood transfusion committees (HTCs) [20], and blood product alternatives such as autotransfusion [21] or unbanked directed blood transfusion [22]. Separately, these efforts have limited impact on the blood supply, often impaired by broader consideration of policy, systems, or environmental factors [23, 24]. Therefore, we will instead use a multifaceted approach to explore gaps and identify solutions under the policy, systems, environment (PSE) change framework [25, 26]. This framework will cross the blood system continuum, in a diverse set of socioeconomic settings and clinical pathways in Kenya. This protocol describes an innovative study that will elucidate barriers to access of safe blood for transfusion and identify PSE interventions through a mixed-methods approach as well as process mapping and modeling of the Kenyan blood system continuum.

## Study aims and hypotheses

This study has three Aims:

### Aim 1a

To use qualitative methods to identify current deficits in the blood system continuum, as recognized by stakeholders in non-urgent, urgent, and emergent clinical pathways across the three socioeconomic settings, and attain an in-depth understanding of the current collection, processing, delivery and use of blood from the perspective of stakeholders across the community, and health and blood banking systems.

### Aim 1b

To quantify the current met and unmet need for blood transfusion in the three different socioeconomic settings and three clinical pathways, and to use these data to characterize the commonalities and differences across settings and pathways that may play a role in the implementation of PSE interventions proposed by stakeholders.

### Aim 2

To define critical components and processes in the blood system continuum subject to impactful interventions that facilitate matching supply with demand in a cost-efficient fashion along two dimensions: location and timing, and build a model representation of the existing network structure for collection, delivery, and demand for blood for transfusion that reflects different sources of supply and demand with their associated socioeconomic and clinical characteristics.

### Aim 3

To select potential future PSE interventions to optimize blood availability through active participation of stakeholders across the blood system continuum in each socioeconomic setting. Interventions will be selected through a combination of a review of interventions in the field, stakeholder input (Aim 1a), quantitative understanding of deficits (Aim 1b) and simulated understanding of potential impact (Aim 2). Ensure selected interventions are both context-appropriate and supported by local stakeholders in the community, health, and blood systems.

## Methods

### Overall approach

**Fig. 1:**
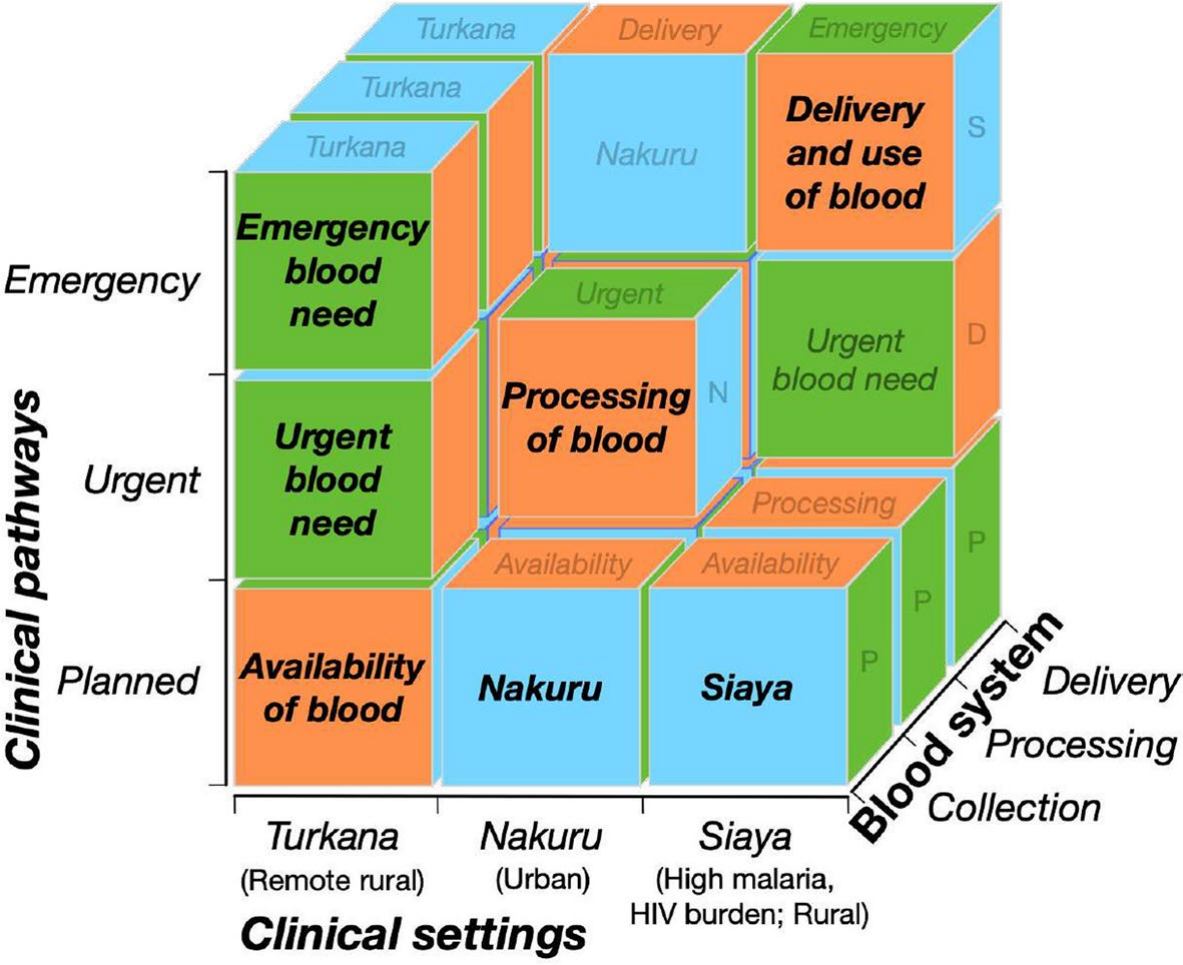
Conceptual model. The conceptual research model encompasses three components of the blood system continuum (collection, processing, and delivery) across three clinical pathways defining blood need (planned, urgent, and emergent), and three socioeconomic settings for care delivery (Nakuru, Siaya, and Turkana). Abbreviations: HIV: Human immunodeficiency virus

This study uses a multidimensional three-by-three-by-three matrix, to facilitate a systematic approach to collecting a broad range of data on the blood system continuum in Kenya across multiple contexts (Fig. 1). Specifically, the study will be performed in three socioeconomic settings: Turkana, Nakuru, and Siaya counties. Turkana County in the north-west of Kenya is characterized by its vast, semi-arid geography and a predominantly pastoralist population. The sparse, widely dispersed population and minimal transport and healthcare infrastructure present challenges for collection of blood and distribution of blood products. The relatively densely populated Nakuru County, in the center of the country, is home to Nakuru City, Kenya’s 4th largest urban center. The wide availability of healthcare services and a high incidence of road traffic injuries in the region presents a context of high demand for blood for transfusion. Siaya County, in the west of Kenya along Lake Victoria, is a rural and densely cultivated region. HIV and malaria prevalence in the population is significantly higher than national averages, affecting donor availability [27], and severe anemia in adults and children present a high demand for blood [28].

In each county, we will investigate the availability of blood for transfusion along three clinical pathways representing the urgency with which blood is needed: emergent, urgent, and planned. Emergent is understood as the clinical scenario where blood need should be met immediately as in the case of critically injured patients or in obstetric emergencies. The urgent pathway refers to acute blood need, not known in advance, that should be met within a few days, such as cardiac decompensation as a result of severe anemia. Lastly, the planned pathway includes clinical scenarios where blood need is known in advance (e.g., elective surgery or planned administration of chemotherapy).

This study will have a systematic approach to modeling the blood system continuum built on understanding gained through qualitative and quantitative data analysis. The industrial engineering (IE) approach includes process mapping (a tool widely used in chain supply, services, and manufacturing industries) as the first step of the process improvement effort that will inform a discrete-event simulation model (DES). The DES model uses the process maps to identify the primary sequence of steps in the process of procuring, storing, and transfusing blood. These are then translated into a computer simulation model that can simulate the process over time. DES models have been utilized to model a broad variety of systems and events, including disaster responses and healthcare systems. Process maps and mind maps, along with simulation models, will allow for virtual perturbations of the system and testing of “what-if” scenarios. Eventually, the integration of the three study methods (quantitative, qualitative, and IE modeling) will allow us to identify and evaluate the potential impact of context-appropriate PSE changes to the Kenyan blood system continuum.

### Study design

This study will be conducted as part of a research program funded by the National Heart, Lung, and Blood Institute (NHLBI) in the National Institutes of Health (NIH) called “BLOODSAFE: Research to enhance availability of safe blood for patients with severe anemia and hemorrhagic conditions in LLMICs in Sub-Saharan Africa” [29]. Studies in this NHLBI-NIH program are designed in two phases: the assessment (UG3) phase, and an implementation (UH3) phase [30]. The focus of the two-year UG3 phase is the assessment of core challenges associated with current blood transfusion services in Sub-Saharan Africa using descriptive epidemiologic approaches, and the development of strategies to enhance the availability and delivery of safe blood for transfusion. The four-year UH3 phase will involve implementing identified PSE strategies aimed at improving the safety and availability of blood transfusion and evaluating the strategies using the Consolidated Framework for Implementation Research (CFIR). The study protocol described herein is limited to the UG3 phase of the program.

### Study period

The study will be carried out over a two-year period. Qualitative activities related to Aim 1a, including data collection and analysis, will take place during the first year. Prospective quantitative data collection and analysis, Aim 1b, will be conducted during the second year. Aim 2 (process mapping and modeling activities) will be performed continuously through the two years, as this Aim will use data acquired through both quantitative and qualitative approaches. Preliminary results from Aims 1a, 1b, and 2 will be used to deliver on Aim 3 towards the end of the second year.

### Data sources

Qualitative data collection for Aim 1a through semi-structured interviews will be divided into three sets. First, key informant interviews will be conducted in each of the three socioeconomic contexts (Turkana, Nakuru, and Siaya Counties) with health system administrators at the national and county levels, including National Ministry of Health officials, County Health Directors, Hospital Administrators, and Nursing Leads from eligible healthcare facilities. These interviews will allow us to gain understanding of their perspectives on the blood transfusion system in Kenya, including system-level challenges and resources necessary for ensuring an adequate supply of safe blood for transfusion (Table 1). The second data collection set will include one-on-one stakeholder interviews with targeted questions to probe for potential deficits, changes, or interventions in blood availability, processing, and delivery. Stakeholders invited will include laboratory personnel, blood bank directors, clinicians (consultants, medical officers, and clinical officers, including both inpatient and outpatient providers), nurses, blood donors, and patients. Lastly, the third data collection set will involve focus groups with stakeholders to maximize data collection through group synergy that promotes exploration of divergent opinions, with special focus on data that are crosscutting across the three clinical pathways. Focus group discussions of at least 6 participants will be held separately for medical officers, clinical officers, inpatient and outpatient nurses, and blood donors to inquire about their perception of the blood system continuum as well as encouraging participant interactions that lead to proposals of interventions.

**Table 1:** Interview guide matrix

**Topic**	**Individual interviews**					**Focus group discussions**			
	Health system, hospital and nursing administrators	Blood bank administrators	Laboratory personnel	Consultants	Patients	Blood donors	Medical officers	Clinical officers	Nurses
**Blood system**									
General perception	✓		✓	✓	✓	✓	✓	✓	✓
Barriers/Facilitators	✓		✓	✓	✓	✓	✓	✓	✓
Innovations/Adaptations	✓		✓	✓	✓	✓	✓	✓	✓
**Hemovigilance**									
Supervision	✓	✓		✓			✓	✓	✓
Reporting	✓	✓		✓			✓	✓	✓
Improvement	✓	✓		✓			✓	✓	✓
**Blood sourcing**									
Donation		✓	✓		✓	✓			
Collection		✓	✓						
**Blood processing**									
Transportation		✓	✓						
TTI testing		✓	✓						
Blood storage		✓	✓						
**Blood requisition process**				✓	✓		✓	✓	✓
**Blood delivery**									
Availability at point of use		✓	✓	✓	✓		✓	✓	✓
Transfusion		✓	✓	✓	✓		✓	✓	✓
**Blood need estimates**			✓	✓			✓	✓	✓

The research team in charge of qualitative data collection will consist of nine individuals, or three teams with a senior researcher, knowledgeable with experience in qualitative data collection in Kenya, and two research assistants. Interviews will be in-person and conducted in English with translators present for Swahili, Turkana, or other local language. Interviews are expected to take between 30 to 60 min with focus groups being slightly longer, up to 90 min. Interview and focus groups discussion guides for the three data collection sets will be reviewed in an iterative fashion to build on new information learned from data gathered in previous interviews and focus groups (Additional file 1). In order to change discussion guides, all researchers involved in qualitative data analysis will need to agree on appropriateness of changes based on interviewee’s feedback and need for further probing.

**Fig. 2:**
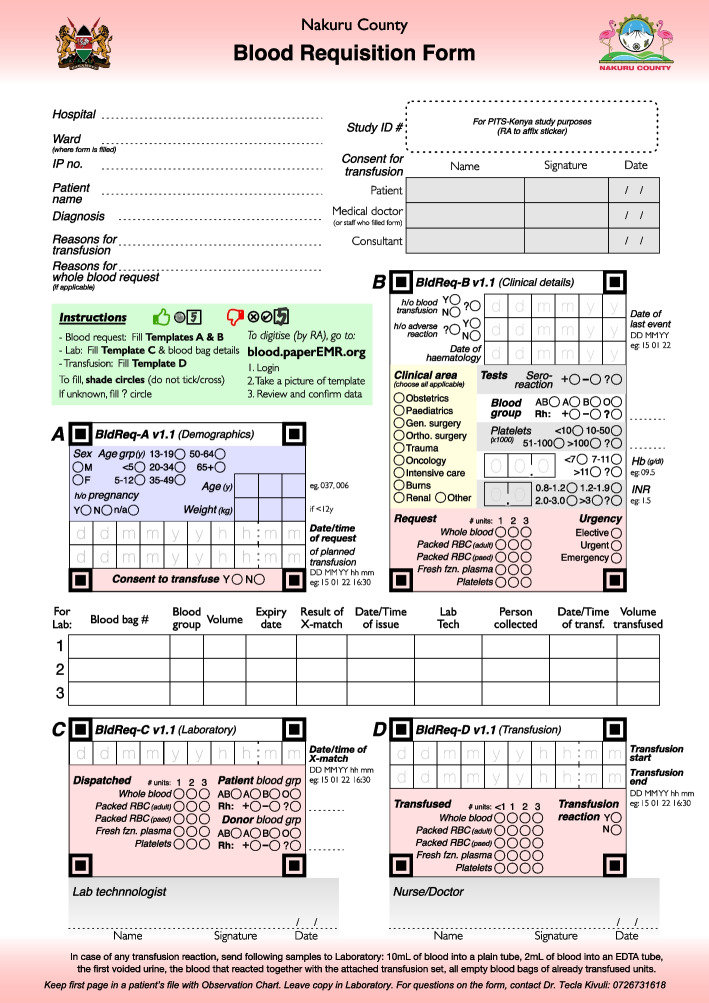
Blood requisition form for data collection. Abbreviations: IP: inpatient, h/o: history of, RBC: red blood cells, Hb: hemoglobin, INR: international normalized ratio, X-match: crossmatch

Prospective data gathering for Aim 1b will include monthly reports currently used by selected blood banking facilities containing information on blood collection logistics, blood donors’ characteristics, testing for TTIs, preparation of blood components, number of blood products requested and dispatched, and current stock. In addition, active data collection will take place at the main referral hospital in each County (Turkana, Nakuru, Siaya) using standardized blood requisition forms (Fig. 2). The forms replicate the current processes for ordering blood by clinicians, cross-matching and dispatching blood in the laboratories, and documenting transfusion in the wards, but allow documented information to be digitized by study personnel using the PaperEMR Web Application, a data collection tool designed for low-resource settings without electronic medical records [31]. This tool will digitize blood requisition, dispatch and transfusion data by reading-in pictures of the form including the unique study identification number assigned to the patient.

The information necessary for process mapping, modeling, and solution development (Aim 2) will be obtained through a combination of focused interviews, blood collection and usage estimates, and direct observation. Specifically, Aims 1a and 1b will gather necessary qualitative and quantitative data on collection, testing and processing, storage, and distribution of blood to inform the analyses. Data sources will include bank records, blood requisition forms, interviews with administrators, clinicians, patients, and donors. Additionally, direct observation of practices will take place at the main referral hospitals, regional blood transfusion center and satellite collection centers involved in the blood supply system in Nakuru, Siaya and Turkana counties.

Preliminary results from qualitative and quantitative data analyses, and process mapping and DES simulation modelling efforts will be used to conduct stakeholder workshops. These workshops are expected to enable active stakeholder participation from across the blood system continuum in each socioeconomic setting the study is targeting. The goal of the workshops is to provide participants with information to debate and guide the selection of context-appropriate interventions to improve blood availability that can be tested using implementation science approaches.

### Study sample

In Aim 1a, we will use maximum variation, stratified, purposive sampling of stakeholders within each of the three socioeconomic settings (Turkana, Nakuru, and Siaya Counties). Purposive sampling has been widely used in research on the blood system continuum [20, 32, 33]. There will be fifteen initial sampling frames, or categories of stakeholders, per socioeconomic setting. Sampling frames will include the following: national health system administrators, county-level health system administrators, hospital administrators of facilities level 3 under the Kenya Essential Package of Health (KEPH) [32], hospital administrators of facilities level 4 or higher, nursing administrators of facilities level 4 or higher, laboratory staff of facilities level 4 or higher, directors of regional blood banks, directors of blood collection centers, directors of satellite blood transfusion centers, medical specialists (consultants) for each of the three clinical pathways (emergent, urgent, and planned need for blood transfusion), medical officers, outpatient clinical officers, inpatient or outpatient nurses, blood donors, and patients for each of the three clinical pathways. Consultants will include those ordering blood transfusions, such as obstetrician and gynecologists, general surgeons, pediatricians, and, if available, intensive care specialists and oncologists. In case of consultants not being available, we will interview other consultants or medical officers with similar blood transfusion responsibilities for that particular socioeconomic context and clinical pathway. This scenario is expected to happen in lower-level public healthcare facilities and blood banks.

A combination of key informant interviews and focus groups will be conducted based on stakeholder types and clinical pathways. A total of 74 individual interviews and 15 focus groups will be performed. Specifically, 3 donor focus groups, 4 focus groups each of medical officers, clinical officers, and nurses. In addition to 8 interviews with national and county-level health system administrators, 12 interviews with facility administrators (medical, nursing, and laboratory), 9 interviews with blood banks administrators, 18 interviews with medical consultants, and 27 patient interviews. This sample size is based on the healthcare system and social context of the Kenyan settings as well as the complexity of blood transfusion across socioeconomic settings and clinical pathways. However, we will evaluate the need of continuing recruitment of participants based on thematic saturation upon iterative analysis of the data and recognizing the lack of new themes.

The sample for Aim 1b will consist of one healthcare facility and one blood bank from each of the three socioeconomic settings. Eligible private and public healthcare facilities include sub-County and County hospitals, facilities level 4 to 5 of the KEPH classification, respectively [34]. Prospective data collection at these facilities will include all blood requisition forms issued within a 10-month period. Data collection will also comprise monthly reports on blood collection and processing for a similar period of time. Regional blood transfusion and satellite blood collection centers are both considered eligible.

Since the objective of the IE approach of Aim 2 is to visually describe the flow of work, it does not require any prespecified sample size. For the purposes of this Aim, participants from healthcare facilities and blood banks will be chosen from among those individuals responsible for the collection, testing, processing, storage, and distribution of blood for transfusion recruited for Aim 1a. Similarly, a selection of stakeholders interviewed for Aim 1a (at least one from each stakeholder category in each socioeconomic context) will be invited to workshops under Aim 3.

### Measures

The qualitative data gathered in Aim 1a will be measured at the code, sub-theme, and theme levels. Each code will represent a construct or idea based on word and phrase repetitions from interviews and focus groups transcripts [35, 36]. Subsequently, categorization of codes will lead to the identification of themes and sub-themes that encompass: the identification of ground level deficits and challenges in the blood system continuum; and the type and characteristics of interventions proposed by stakeholders at the policy, system, and environmental level addressing identified critical challenges. There will be emphasis on identifying barriers and facilitators to potential implementation of PSE interventions as well as the differences and commonalities across clinical pathways and socioeconomic settings.

In Aim 1b, monthly reports from selected blood banks will be interrogated for data on blood collection and processing including: blood collection targets; blood collection by type of donor (VNRBD or FRD) and blood type; blood donors rejected and reasons; blood products requested; and blood products dispatched. Data from digitized blood requisition forms will include the following variables recorded by clinicians: patient demographics; patient diagnosis and indication for transfusion; history of blood transfusion; hospital ward and clinical pathway; patient blood group and blood tests including hemoglobin level; and number and type of blood products requested. Laboratory personnel will provide data on the number and type of blood products dispatched, and cross-matching testing date and time. Lastly, clinicians will record the number and type of blood products transfused to the patients as well as the time transfusion started and ended, and the presence of blood transfusion reactions.

The IE approach (utilizing process mapping and modeling) of the Kenyan blood banking system in Aim 2 will make use of demand and supply for blood for transfusion in each of the three socioeconomic contexts developed in Aim 1b. Also, it will include characterization of each individual step of the blood system continuum. Description of the collection step will focus on donor recruitment and donor screening. Moreover, the testing and processing step will be described in terms of TTI prevalence, testing capacity, and testing strategies. Storage and distribution will mainly evaluate human resources, coordination and transshipment policy, and logistics. Finally, information on clinical transfusion protocols and technologies will be included in the transfusion step. The stakeholder workshops under Aim 3 are expected to result in prioritization of context-specific interventions in each socioeconomic setting.

### Analysis

All interviews and focus group discussions in Aim 1a will be audio recorded. They will be transcribed verbatim using Otter.ai™ software and verified for accuracy [37]. For certain groups of respondents such as patients and blood donors, translations of transcripts will be done from local languages (Swahili, Luo, and Turkana) to English. Interviewed participants will be de-identified by blinding to the specific demographics of each interview subject but include the data necessary to identify the sampling frame. We will use a thematic analysis approach on all transcribed data to identify themes, starting with general coding and then including more specific codes as data analysis proceeds and researchers develop and refine a working model for the relationships within the data using MAXQDA 2022™ software [38]. Codes are not pre-set, and a codebook will be developed through an inductive process based on an iterative review of the transcripts.

Transcripts will be coded by at least two independent coders analysts who were not involved in conducting the interviews. To ensure validity and reliability at least two investigators will code separately using thematic analysis and constant comparison of data. Iterative analyses of collected data will be held to assess whether thematic saturation has been reached. Similarly, group discussions will be held among the research team to review identified themes and adjudicate coding disagreements. At this iterative assessment points, the research team will determine if further interviews are required. Lastly, qualitative data analysis will be complete when representative themes have been identified regarding variations, challenges, and potential solutions for safe and timely blood availability, processing, and delivery across the three socioeconomic settings and levels of urgency of blood transfusion [35, 36]. Qualitative results will be presented as a summary of themes, categories, and sub-themes including individual segments of quotations representing them.

For the quantitative data collection in Aim 1b, All blood bank monthly reports will be captured into a single dataset for each of the selected blood banks. Similarly, digitized blood requisition forms will be entered into one database grouped by healthcare facility. The analysis of quantitative data will include statistical description of monthly reports and blood requisition forms at each selected blood bank and healthcare facility as well as statistical comparisons across socioeconomic contexts and clinical pathways. Descriptive analysis of monthly reports from each blood bank will provide information on blood collection targets, frequency, and proportion of blood collection by donor type (VNRBD vs. FRD), total units of blood collected by blood type, blood donor rejection rates and most common reasons. Data from blood requisition forms in each hospital will be analyzed to provide monthly estimates on at least the following three aspects of the blood delivery process: frequency of mismatch between the number of blood units requisitioned by clinicians and the number released by blood bank; time elapsed between blood requisition and transfusion; and frequency of mismatch between the number of blood units released by the blood bank and the number transfused. These values, in turn, will be compared across the three socioeconomic contexts and three clinical pathways. Results will also include quantitative data on blood donation, requisition, and delivery to inform the process mapping and modeling activities of Aim 2.

Analyses for Aim 2 will include patient-centered process mapping of the blood system continuum as well as modeling and solution development, where patients generate the demand for blood for transfusion and then exit the process map following treatment. First, blood supply and demand will be considered as a partial pull-based process centered around patients, where need for blood for transfusion is the primary input and patient reception of transfusion is the primary output. We will visually depict the process between the primary input and the primary output through a process map. It will include characteristics such as: sequence of steps, inputs and outputs, resources needed, estimated times, decisions to be made and actions to be taken, and performance measures. Overall, the process map will summarize how demand arises; how the schedule for meeting this demand is determined; how blood is sourced, transported and stored; what associated supplies are required and how these are sourced, transported and stored; how decisions are made on allocating blood to patients; what happens when either blood for transfusion or other resources are unavailable for the patient; and what specific steps the patient goes through before exiting the blood system continuum. More importantly, this will allow us to identify inefficiencies or bottlenecks to eventually inform the development of appropriate interventions to address and mitigate these shortcomings.

Then, we will develop analytical methods to create hypothetical scenarios of disruptions to the blood system continuum based on the process mapping results. Multiple models including deterministic relationships and DES simulation models will be developed. Possible model parameters will include donor recruitment strategies and processes, number of units collected per strategy, likelihood of repeat donation, conversion from family replacement to voluntary donor, prevalence of TTI, testing and processing capacity and infrastructure, demand for specific products, storage capacity and infrastructure, transportation infrastructure, health system priorities (implicit/explicit prioritization of clinical conditions), clinical demand for blood, transfusion practices, transfusion protocols, time spent in each step of the protocol, and deviations from the protocol.

Each method under this protocol is intended to provide a lens to view the blood system continuum. But the combination of approaches is likely to deliver insights that each method by itself does not. The integration of the three methods (qualitative, quantitative, and DES simulation modelling) is aimed at guiding the selection, implementation, and testing of one or more interventions aimed at increasing the availability of blood.

Improvements to the blood system continuum through PSE changes will be estimated as a model response to the interventions in each step of the process (collection, processing, delivery and use). For instance, changes to “collection” may result in an increase in the number of voluntary blood donors in each drive, optimized frequency of blood drives, or reduced periods of blood product surplus and deficit. These in turn could influence, positively or negatively, steps of processing, distribution and use. Similarly, interventions in “processing” could be represented as reduction in time elapsed between collection and use of blood, and in blood waste. Changes to “delivery and use” could reduce blood inventory losses, reduce storage and operational costs, or improve turnaround times for transfusion procedures. Simulated results will therefore inform the selection of PSE changes by stakeholders.

## Discussion

Given the complexity of the blood system continuum, and the diverse array of stakeholders involved at various points in the process, a mixed methods approach to the Kenyan blood transfusion system is appropriate. This study design combines quantitative and qualitative research methods with IE approaches to understand and assess challenges to the availability of safe blood for transfusion. In addition, the unique study three-by-three-by-three design allows for the comparison of findings across different socioeconomic settings and varied clinical needs for transfusion within one country. The findings of this mixed-methods study will provide a comprehensive understanding of the blood system continuum in Kenya, and the evidence to support the selection of contextually-appropriate interventions to meet the blood transfusion needs of the population. This understanding will directly contribute to the conduct of future implementation studies – the design of interventions and the approaches to measure their impact.

The three study sites selected for this study (Nakuru, Siaya, and Turkana counties) represent widely varying contexts for healthcare delivery, ranging from remote rural to urban, and with different local prevalence of TTIs among blood donors and differing disease burdens in the population. Similarly, blood is needed in the management of diverse conditions and along different clinical pathways. The processes involved in ensuring blood availability for the management emergencies (e.g. rapid, citizen-led mobilization of donors in response to emergencies like terrorist attacks) vary from those involved in ensuring blood availability for planned surgeries or chemotherapy. The study approach therefore recognizes that the blood system continuum may work differently across diverse socioeconomic and clinical conditions. In fact, understanding these differences in healthcare and blood banking may not only facilitate the design and implementation of interventions but also further research into health inequities in the global blood supply.

In addition, we emphasize the importance of stakeholder identification of potential interventions and subsequent characterization in terms of policy, systems, and environment changes [39]. The PSE change framework contributes structures for strategic and systems-thinking, partner engagement throughout the process, and decision-making structures for planning, implementation, institutionalization, and expansion of learning [25, 26]. This study applies a systems-approach uniquely focused on “availability of blood for transfusion at the point-of-use”, the sharp end of the care setting. Using a person-centered approach, the design also allows for direct measures of blood ordering and availability contributing to timely blood transfusion at the bedside. Further, the multi-disciplinary perspectives from those faced with closing the gap on unmet or delayed blood transfusion needs will contribute new insights to areas less described in the current literature. Importantly, the action learning approach invites innovations and adaptations to be examined within the context of the problem, informing what changes may have the greatest impact on time, quality, and cost and ultimately contributing to improved blood transfusion availability.

The research team contributes an extensive array of expertise, such as trauma, surgery, critical care, obstetrics and gynecology clinicians, blood banking and blood transfusion experts, health systems experts, public health practitioners, and industrial engineers within a geographically diverse team. While the application of quantitative methods characterizes the variables, gaps, and opportunities; the qualitative research contributes explanatory meaning to gain a deeper understanding of the context and challenges presented in this real-world setting. The abstraction of the problem into a patient-centered supply model allows for modulation of myriad variables and interventions to optimize the desired outcome. The diversity of the team and the range of expertise contribute to a rich milieu of contextualized innovation and entrepreneurial insights to address the problem of blood availability at point-of-use under a PSE change approach.

This study has several limitations. First, the results obtained may need active knowledge transfer between research team members and stakeholders. Integration of data collection and results across the three aims may prevent the outcomes of this study from being easily compared to other studies where these same methods were used alone. Given the scope of the effort, the sampling of stakeholders for the qualitative study is purposive, to ensure representation of the different stakeholders. This is not sufficient to ensure in-depth exploration of specific topics, for example, barriers to blood donation. Similarly, the quantitative data collection focuses on major hospitals of each county. While this may provide an estimate of overall met (and unmet) needs in each county, significant additional effort will be needed to obtain national estimates of blood need. This approach is geared primarily towards the identification of context-specific interventions that can be implemented to improve availability of safe blood for transfusion in Kenya.

The importance of contextualized interventions in blood supply cannot be overstated. In these settings, nutritional status, beliefs, education and awareness around blood, geographic, financial and other barriers to access healthcare facilities, the burden of trauma and other diseases, are all likely to differentially influence the demand for blood transfusion. These same conditions reduce the proportion of the population that are suitable candidates for blood donation. In addition, attributes of the health system such as governance, financing, information and procurement systems, human resources and supply chains affect the availability of blood and the ability to intervene along the continuum. Our study aims to grapple with this complexity, using a diverse set of approaches and tools, to develop a holistic understanding of the blood system continuum in LLMICs settings, and detail the context in which any intervention is attempted.

The overall goal of the research team is to improve the availability of blood at the point-of-use. Interventions towards this could include range from distributive models for blood safety testing, innovative techniques for patient blood conservation, or community-centered participatory frameworks that encourage social solidarity surrounding blood donation and availability. While interventions are likely to vary depending on context-specific needs, their success is likely to depend on active inclusion and participation of all stakeholders to navigate a complex system. Learning from the application of these interventions could be informative to other settings faced with similar challenges.

### Supplementary Information

**Additional file 1.**

## Data Availability

Not applicable. No data have been collected yet.

## Abbreviations

CHMTs: County Health Management Teams
FRD: Family Replacement Donation
HTC: Hospital blood transfusion committee
KEPH: Kenya Essential Package of Health
KNBTS: Kenya National Blood Transfusion Service
LLMICs: Low and Lower-Middle Income Countries
NHLBI: National Heart, Lung, and Blood Institute
NIH: National Institutes of Health
PEPFAR: United States President’s Emergency Plan for AIDS Relief
PSE: Policy, System, and Environment
RBTC: Regional blood transfusion center
TTIs: Transfusion transmissible infections
VNRBD: Voluntary Non-Remunerated Blood Donation
WHO: World Health Organization
