# Supplementary material for: Protocol: identifying policy, system, and environment change interventions to enhance availability of blood for transfusion in Kenya, a mixed-methods study
Source: BMC Health Serv Res. 2023 Sep 7;23:963. doi: 10.1186/s12913-023-09936-0 (PMC10486046; doi:10.1186/s12913-023-09936-0)
Supplement: Supplementary file 1 — Additional file 1. [file 12913_2023_9936_MOESM1_ESM.pdf]

PITS Qualitative Tools

Tool 1a: Individual Interviews with National stakeholders

Introductory Prompt

Thank you for agreeing to meet with us. We are interested in understanding how the blood system in Kenya works, ranging from blood collection to processing, delivery and use of blood. Through these interviews we hope to gain perspective on current barriers and possible solutions.

<u>Questions</u>

1. **State of the blood system in Kenya**
    - What is your role with regard to blood transfusion in Kenya?
    - What is your opinion on the current status of blood transfusion in the country?
        -
        - **Probe**: What is working well ? What is not working well?

2. **Blood Donation and collection**
    - The main sources of blood are either volunteer donations through drives, or family replacement donors. Is the country prioritising blood donation from either of these two sources?If so, how?
    - Is your organisation involved in recruiting blood donors? If yes, how? Who else is involved? How are donors motivated (by you or other partners)?
    - Does your organisation participate in organising or conducting blood drives? If yes, how? Who else is involved in the blood drives you participate in?
    - Does your organisation track blood donors, or do you know of efforts to track blood donors? If so, can you tell us more about managing relationships with donors?
    - What do you see as some of the barriers to increasing the number of blood donors in Kenya?
        - **Probe:** Do you see cultural barriers/misconceptions about blood transfusion, or logistical challenges to donation, etc.

3. **Blood screening, processing and storage**
    - Can you broadly describe the processes involved in screening blood for transfusion transmissible illnesses (TTIs), and processing into components, in Kenya?
    - Are you aware of any challenges in screening of blood for TTIs?
        - Probe: Can you describe some?

- Are you aware of any challenges in processing blood into components?
    - Probe: Can you describe some?
- Are you aware of systems for maintaining quality in screening and processing of blood?
    - Probe: Are you aware of any challenges with quality systems?

4. **Blood distribution and use**
    - Can you broadly describe how blood or blood components are distributed to the facilities where they are needed?
        - **Probe:** Are you aware of any challenges in distributing blood or blood components to where they are needed?
    - Are you aware of any systems to track the availability and distribution of blood in Kenya?
        - **Probe**: If yes, can you describe any?
        - **Probe**: Has your organisation invested in any system/technology for tracking the availability and distribution of blood?
    - Do you think the current requisitions made for blood at public hospitals reflect the true need for blood within the county?
        - Probe: do you think clinicians request more or less blood than what they need? Why?
    - Are you aware of any challenges to how blood is used by healthcare providers?

5. **Supervision and oversight of blood transfusion**
    - Does your organisation receive any reports on the state of the blood system in the country or specific county?
        - Probe: e.g. reports on blood units collected, processed, distributed, or used?
        - Probe: If yes, how do you use the information you receive on the blood system?
    -
    - Does your organisation provide any information, advice or feedback to National or County health administrations?
    - Does your organisation influence decisions or policies on blood transfusion made at the national level? If so, how?
        -
    - What actions does your organisation take, if any, in response to shortages of blood at hospitals or at blood banks?

6. **Resources for blood transfusion services**

i) Funding of blood drives

- Does your organisation **finance** any steps of the blood system (collection/screening/distribution), and if yes, how?
    - Probe: What is the scale of the financing ?
    - **Probe:** Do you provide any non-financial support?
- Who are your partners involved in supporting the blood system?
    - **Probe**: What role do they play? (technical/funding)
    - **Probe:** What is the scope of their support ?

ii) Private sector Partnerships

- Does your organisation work with private sector entities to support the blood system in the county?
    - Probe: If yes, can you give an example of such organisations and how they are involved? If not, what support do you think the private sector can provide?
    -

8. Supervision and improvement mechanism

- What do you perceive as the main challenges to the blood system in your county?
    - Probe: how could they be overcome?

Tool 1b: Individual Interviews with County Health Leadership

Introductory Prompt

Thank you for agreeing to meet with us. We are interested in understanding the blood need in your region/health facility and understanding how blood availability, processing, and delivery works. Through interviews we will be gaining perspective on current barriers and possible solutions.

<u>Questions</u>

1. **State of blood availability in the County**
    - What is your role with regard to blood transfusion in the County ?
    - What is your opinion on the current status of blood transfusion in your county?
        - **Probe**: In the Country?
        - **Probe**: What is working well ? What is not working well?
    - Would you say that your county receives sufficient blood to meet its blood transfusion needs?
        - **Probe**: If not, what does the County do to ensure that the unmet need for blood is met ?

2. **Blood Donation and collection**
    - The main sources of blood are either volunteer donations through drives, or family replacement donors. Is the county prioritising blood donation from either of these two sources?If so, how?
    - Does the county get involved in recruiting blood donors? If yes, how? Who else is involved? How are donors motivated (by you or other partners)?
    - Does the county participate in the organisation of blood drives? If yes, how? Who else is involved in blood drives in the county?
    - Does the county track blood donors, or do you know of efforts to track blood donors in the county? If so, how?
    - What are some of the barriers to blood donation by potential blood donors in your county?
        - **Probe :** Cultural barriers/misconceptions about blood transfusion etc.

3. **Blood screening, processing and storage**
    - Can you describe how blood is screened and processed (into components), and stored in your county?
    - Are there sufficient resources (human resources/equipment/funding) for blood screening and processing and storage?

    - **Probe**: If not, what additional resources might be required for screening and processing ?
- Are you aware of any challenges in blood screening and processing in your county?If there are, can you describe them?
- Are you aware of any ways quality is maintained in the screening and processing of blood in your county?

4. Blood utilisation and distribution

- What is your opinion on the current utilisation of blood within your county?
- Do you think the current requisitions made for blood at public hospitals reflect the true need for blood within the county?
    - Probe: do you think clinicians request more or less blood than what they need? Why?
- Can you describe How blood /blood components are distributed to where they are needed within your county?
    - **Probe:** What transport resources would be needed?
- Are you aware of any system or policy to track blood and blood components within hospitals in your county ?
    - **Probe**: is it paper/technology/mobile phone?
    - **Probe**: Have any hospitals within your county invested in any system/technology for tracking blood/components?
- Does your county have hospitals that have an inventory management policy for blood and blood components?
- What are some of the challenges you perceive to the distribution of blood within your county?

5. Reporting and policies on blood transfusion

- Do you receive any reports on the state of blood transfusion in your county?
- As part of the County Health Management Team (CHMT), how do you use the report you receive to improve blood transfusion services?
- Describe the kind of feedback you provide to the County Referral Hospitals after reviewing the monthly report on the blood transfusion system,
- Do you influence blood transfusion decisions and policies made at the national level? If so, how ?
    - **Probe:** Are you able to provide information to the National level that assists them with decision making /policies on blood transfusion?
- What actions have you undertaken at the management level when there is a shortage of blood at hospitals or at the Blood bank within your county?

6. Resources for blood transfusion services

i) Funding of blood drives

- Does the county **finance** aspects of blood transfusion (sourcing/screening/distribution), and if yes, how?
    - What is the scale of the financing ?
    - **Probe:** equipment, drives, screening, distribution
- What is the role of the partners who are involved in supporting the blood transfusion system in your county
    - **Probe**: What role do they play? (technical/funding)
    - **Probe:** What is the scope of their support ?

ii) Human resources

- Does your County have sufficient health care workers for managing the blood transfusion process ? If no, what kind of support does the county provide specific to the human resources involved in the blood transfusion process?

7. **Partnerships**

- Does your county work with private sector organisations to support the blood needs of the county?If yes, can you give an example of such organisations and how they are involved?If not, what support do you think the private sector can provide?
    - **Probe:** For example, have you been approached by or work with organisations like Lifebank/Damusasa?

8. Supervision and improvement mechanism

- How does the National Government supervise County activities with regard to the blood transfusion process?
    - **Probe:** Is there anything your County is doing to improve blood donation/processing/distribution/storage/transfusion?
- What do you perceive as the main challenges to the blood system in your county? Probe: how could they be overcome?

Tool 1c: Individual Interviews with Medical and Nursing Administrators

Introductory Prompt

Thank you for agreeing to meet with us. We are interested in understanding the blood need in your region/health facility and understanding how blood availability, processing, and delivery works. Through interviews we will be gaining perspective on current barriers and possible solutions.

Questions

1. **Role in blood logistics**
    - What is your role with regard to the blood transfusion process at your health facility?
    - What is your impression on the state of **blood availability** in your health facility and at the County?
    - Can you describe a recent clinical scenario where you were involved in making blood available for a patient at your health facility, kindly explain what happened?
    - What is your impression on the state of **blood delivery** in your health facility/ at the County?

2. **Blood processing**
    - Can you describe the steps involved in ordering and receiving blood in your health facility **from the blood bank**?
        - **Probe:** If your health facility is situated at a different location from the blood bank , does this process differ ?
    - If you have worked in different hospitals (larger or smaller facilities, or in the private sector), have you seen differences in how blood is ordered and received at those hospitals?
    - Which blood products do you normally transfuse to your patients at your health facility?
    - Is there a greater need for other blood products/components other than whole blood e.g packed red cells, platelets? Fresh Frozen Plasma?
        - **Probe :** Do you process the blood products at your health facility or do you source them externally?
    - If you source them externally are there any challenges with getting the blood products on time when your patients need them ?
    - Do you have the necessary equipment for processing the blood components ?
    - Do you experience any delays with receiving blood products at your health facility?

3. **Blood Prioritisation**
    - What are the most common conditions for which blood transfusions are needed in your health facility?
    - How does prioritisation of blood happen at your health facility ?
        - **Probe :** What role do you play in the prioritisation of blood at your health facility ?
    - What happens when there is a stockout/shortage at your health facility?
        - **Probe** : What do you do, or what steps do you take? For example are additional efforts made to secure blood for patients with some conditions over other patients

4. **Supervision, reporting, errors and improvement mechanism**

- What is your role regarding supervision processes in deciding how to **manage** blood (both within your health health facility and to other health facilities)
    - **Probe :** availability/Distribution/ conflict during a shortage
- Which agencies or organisations are involved in the process of blood transfusion ? and what is their role in blood transfusion?
    - **Probe :** At the County? Other NGOs
- Are resources available for improvement, at national, county or facility level?
- Are the competing priorities for resources for blood transfusion from national and county level
    - **Probe :** e.g resources for conducting blood drives e.g sodas/ allowances/ Sufficient human resources allocation?
- Do you know of any recent approval of new policies/technologies/ standard operating procedures from the KNBTS regarding blood transfusion?
    - If yes, which ones and have they affected the process of blood transfusions at your health facility?
- Do you know of any problems in the blood transfusion system e.g reporting of blood testing, results of TTI screening/cold chain systems/ errors by clinicians during blood transfusion (eg in blood typing/cross matching/transfusion related reactions) etc.
- How does your health facility handle such problems ?
    - **Probe :** Does your health facility have quality controls to ensure the continuity of the cold chain?
- What is your role in the design and implementation of improvement strategies for operations around blood transfusion ?
    - **Probe** : improvements in blood availability/processing/ transportation
    - **Probe** : resources- financial resources/ human resources etc.
    - **Probe:** policies
    - **Probe:** functioning of Blood transfusion committees at the hospital

5. **Clinical Pathways for blood transfusion at your health facility**

We would like to understand how blood is available depending on the urgency with which blood is needed: 1) Emergencies (e.g. trauma or PPH), 2) Urgent conditions (e.g. severe anaemia in children or adults), and 3) Non-urgent conditions (e.g. before chemotherapy or cancer surgery)

- Do processes for ordering blood change if blood is needed in an emergent, urgent or planned situation?
    - **Probe:** If so, how?
- Does your health facility receive enough blood, and in time, to manage these different types of blood needs?
    - **Probe:** For example, which blood need (emergency, urgent or non-urgent conditions) do you have more challenges meeting, if at all?
- What clinical conditions drive emergencies at your health facility ?
- Are there any additional steps you take in case blood is needed in these emergencies?

6. **Challenges and solutions in blood transfusion**

- What do you perceive as the current challenge with the blood transfusion process
    - **Probe** : blood availability/donation/ processing/ transportation
- What are your proposed possible solutions to improve the blood transfusion process (the availability, processing and/or delivery of blood) ?despite system-wide shortfalls?
- Can you identify facilitating policies to the blood transfusion process that have had a positive impact on blood transfusion?
- Can you identify any policies that are acting as a barrier to the blood transfusion process and have negatively impacted blood transfusions?
    - What are your proposed suggestions towards the policy?

Tool 2a: Individual Interviews with Blood Bank Administrators

Introductory Prompt

Thank you for agreeing to meet with us. We are interested in understanding the blood need in your region/hospital and understanding how blood availability, processing, and delivery works. Through interviews we will be gaining perspective on current barriers and possible solutions

1. **Blood Logistics**

   a. **Role in blood transfusion process**
      i. Can you describe your role in the blood transfusion process?
      ii. What approaches do you use to ensure you obtain blood to fulfil the requests of clinicians from different hospitals?
      iii. Can you describe the most recent clinical scenario where a clinician/hospital needed blood, and what happened?
         - PROBE: How do you ensure clinicians/hospitals obtain the blood they need in time?
      iv. What do you do in the case of a blood shortage?

2. **Blood sourcing and collection**

   i.Can you describe your blood bank's approach to recruiting blood donors?
   - PROBE: How are donors contacted? What locations are used for blood collection?

   ii. What are the different sources of blood donations?
   - PROBE: What happens when blood is sourced from family members ?Is the process of collection different from blood sourced from blood drives and other sources of blood donation?

   iii. Who finances blood donations drives?
   - Are the blood donors ever compensated?
   - PROBE : How do you motivate donors to repeat blood donations?

   iv. Is blood collection *ad hoc* or is there a schedule or is it a combination?
   - Is there any form of tracking of donors?
   - PROBE: Digital/ manual register of donors etc.

3. **Testing and Processing:**

   i. Can you describe how blood is processed at your blood bank? How many personnel do you have to do the testing at the lab?
   ii. How long does testing/processing of blood take once collected from donors?
   iii. What equipment is essential for the proper functioning of the blood bank and does your blood bank have them?
   iv. What laboratory tests do you run at your blood bank?
   v. What resources are required for testing blood collected?
   vi. Is blood separated into its components at your blood bank? And if yes, which ones?
   PROBE: How often are they separated into components? How do you do the separation?
   vii. What delays do you typically experience in this process?
   PROBE: Reagents/Human Resource/(delays related to collecting all tested samples together)

4. **Prioritisation**

I. What type/level of hospitals do you prioritise blood orders for at your blood bank?

- How do you prioritise hospitals for receiving blood?
- Who prioritises blood orders to hospitals?
    - Prompt: How does the system work if blood needs to be prioritised for certain hospitals?
- What happens when there is a stockout/shortage?
    - Prompt : What do you do, or what steps do you take?

5. Blood storage and distribution

i. Who oversees storage of blood at your blood bank?

- Who makes the decision at your blood bank on the distribution of screened/processed blood?
- PROBE: Is there a specific person who signs off on the release of blood from your blood bank?
- Who “owns” blood at each stage?

ii. Where is tested/screened/unscreened blood stored within the blood bank?

- PROBE: Central location/ multiple locations/ within hospitals/clinics?
- What are the requirements for safely storing or transporting blood?
- Does your blood bank have the essential equipment needed for storage?
- PROBE :Does equipment availability or maintenance create a problem, if it does, how so?
- How long can blood/blood components be stored at your blood bank?
- How is “screened/processed blood" delivered from where it is collected to the point of use?
    - What works well with the blood delivery at your blood bank?
- Is there a cost to hospitals for units of blood delivered?
- Does transportation of blood create a problem, if it does, how so?

iii. How are the blood/blood products tracked?

- Do you use any information system or tracking technology? If yes which one
- Do you have any inventory management policy? If yes please describe

6. Blood Supply Estimations

- Currently, approximately how many units of blood do you typically COLLECT in a month?
  Prompt: Would you say it was (<5, 5-10, 10-15, 15-20, >20 units)
    - Probe: Do you get the blood from blood drives/ replacement donors?
    - Probe : How many units do you receive from external sources ?
- About how many times in a month have you ever discarded blood received (from the different sources) and why does this happen?
- In an ideal world, how many units of blood would your lab have in stock at any one time in a month?
- Currently, approximately how many units of blood do you typically RELEASE in a month?
    - Prompt: Would you say it was (<5, 5-10, 10-15, 15-20, >20 units)

- o Probe: are these units released to the hospital/ lower level health facilities?
- Is there a standard procedure for giving different hospitals (such as named below) specific amounts of blood ?
    - o Level 4
    - Level 3
    - o Level 2
    - o Private hospital
    - o Other?

7. Supervision, reporting and improvement mechanisms

- Which agencies or organisations are involved in the whole process of blood transfusion?
    - o PROBE: of these organisations which ones does your blood bank work with closely? And what roles do these organisations play in your collaboration with them?
- Are you aware of any new technologies, operating modes or policy changes within the Kenya National Blood Transfusion Service (KNBTS) ?
- Do you experience any problems, errors or mistakes in the reporting or handling of blood?
- Have you been involved in the design and implementation of any improvement strategies or operations?

8. Challenges/barriers and Solutions

a) What, in your opinion, are the barriers to providing clinicians/hospitals with blood?

- Probe: Issues with donation/availability or processing or delivery

b) What solutions do you see to overcome these challenges?

- Probe : Improvements in donation/availability or processing or delivery

- What do you think the Blood Bank could do to improve blood transfusion services?
- In your opinion, what are some of the ways to improve the blood transfusion system in Kenya?
- What differences exist between a public and private hospital in terms of collection, processing, and storage of blood?
- Have there been any positive or negative changes you have noticed in the blood transfusion system during your career?

Tool 2b: Individual Interviews with laboratory staff (Cross-cutting)

Introductory Prompt

Thank you for agreeing to meet with us. We are interested in understanding the blood need in your region/hospital and understanding how blood availability, processing, and delivery works. Through interviews we will be gaining perspective on current barriers and possible solutions.

1. Blood Logistics

- **Role in blood transfusion process**
    - Can you describe what steps you take to ensure delivery of blood to clinicians ?
        - Probe : Where do you receive blood from?
- **Blood process**
    - When blood is requested from your laboratory,can you describe the steps for an MO/CO to put an order in for blood?
        - Probe: Can you describe the most recent clinical scenario where a clinician needed blood (for their patient) and what happened?
    - How might these steps change in a facility with a blood bank versus one without a blood bank, based on your experience in other health facilities?

2. Blood testing and processing:

- Do you do testing and processing of blood/blood products in your laboratory? Where is it done? If not done at your lab, how do you test/process?
- What are the tests/procedures you perform?
    - Probe : Can you describe what role you play with regard to testing and processing of blood/blood products?
- How long does the testing and processing of blood/blood products take respectively?
- What equipment is necessary for the testing and processing of blood/blood products?
    - (Probe cross-matching)
- What are some of the problems that can be encountered during the testing and processing of blood?
    - (Probe- TTI's/exposed blood etc.)
    - (Probe - blood disposal policy?)
- Elaborate further on what happens in the scenarios described above?

3. **Blood storage**

- How do you store blood/blood products (components)?
    - PROBE : (Blood from different sources e.g blood drive/satellite centre)
- Where are your blood/blood products (components) stored?
- What are storage requirements/specifications?
- What is the capacity of blood/blood products that you can store at your laboratory?
- Do you have all the equipment necessary for the storage of blood and blood products/ components?
- Do you ever have problems with storage of blood/ blood products?

- Probe : Problems with equipment used in storage?
- If so, what equipment requires maintenance? What is needed to get it functional? Who is responsible for maintaining/replacing equipment?

4. **Prioritisation of blood**

- When you receive multiple requests for blood, but you have a shortage, how do you prioritise the distribution of blood to clinicians?
    - Do you have any examples of when this has happened, please describe ?
- Where do you receive blood from?
- If you require more units of blood, what do you do? How do you request this?
    - Probe: Is this a formal or informal process?
- How many units of blood do you keep in the lab at one point in time?
- Is there a standard amount of blood you aim to have available in the lab? (If so, who sets this standard?)

5. Blood Supply Estimations

- Currently, approximately how many units of blood do you typically COLLECT in a month? Prompt: Would you say it was (<5, 5-10, 10-15, 15-20, >20 units)
    - Probe: Do you get the blood from blood drives/ replacement donors?
    - Probe : How many units do you receive from external sources ?
- About how many times in a month have you ever discarded blood received (from the different sources) and why does this happen?
- In an ideal world, how many units of blood would your lab have in stock at any one time in a month?
- Currently, approximately how many units of blood do you typically RELEASE in a month?
    - Prompt: Would you say it was (<5, 5-10, 10-15, 15-20, >20 units)
    - Probe: are these units released to the hospital/ lower level health facilities?
- Is there a standard procedure for giving different hospitals (such as named below) specific amounts of blood ?
        - Level 4
        - Level 3
        - Level 2
        - Private hospital
        - Other?

6. Challenges and solutions

- What are the challenges you face in ensuring availability of blood to clinicians who need blood for their patients?
- What barriers have you faced in securing blood within the hospital?From the blood bank?
- What are the challenges that you have faced in testing and processing of blood/blood components?
- Can you describe some of the existing policies related to testing/delivering/processing/storing blood?
    - Probe :Do you adhere to these standards of practice/policies?
- What are the barriers you face in the transportation and delivery of blood to a patient?

- Do you think your county/region faces any unique challenges?
    - If so, describe some of them to us?

Possible Solutions

- What solutions do you see to overcome these barriers?
- Probe : Improvements in donation/availability or processing or delivery
- Probe : Improvements in the Blood Bank system functioning
- What do you think works well with the blood transfusion system in your hospital?in the County/Country?
- Have there been any changes you have noticed in the blood transfusion system during your career?
    - PROBE: What are the positive and negative changes?
- Are there any best practices in terms of collection, processing, and storage of blood that can provide solutions to the barriers mentioned above?
    - Probe: Nationally/private hospitals
- Out of curiosity have you ever donated blood in the last year?

Tool 2c: Individual Interviews with Consultants

Introductory Prompt

Thank you for agreeing to meet with us.We are interested in understanding the blood need in your region/hospital and understanding how blood availability, processing, and delivery works. Through interviews we will be gaining perspective on current barriers and possible solutions.

1. **Blood Logistics**
    a. **Role in blood transfusion process**
        - Can you describe your role in obtaining blood for your patients
        - What approaches do you use to ensure you obtain blood for your patients?
        - Can you describe the most recent clinical scenario where one of your patients needed blood, and what happened?

    b. **Blood process**
        - How does a clinician put an order in for blood?
            - Prompt: Can you describe the steps?
        - Does this differ between outpatient and inpatient settings?
        - From where do you usually receive the blood?
            - Prompt: For example, from the hospital lab, different hospital, or blood bank or satellite centre
        - If you work (or have worked in different hospitals, have you seen differences in how you order and receive blood at those hospitals?

    c. **Blood prioritisation**
        - What are the commonest types of patients you order blood for?
        - How do you prioritise patients for receiving blood?
        - Who prioritises blood orders?
            - Prompt: How does the system work if blood needs to be prioritised for certain patients?
        - What happens when there is a stockout/shortage?
            - Prompt : What do you do, or what steps do you take?

2. **Clinical pathways for blood transfusion**

We would like to better understand your practice around blood transfusion for the following three clinical situations: 1) Emergencies (when blood is needed in a matter of hours), 2) Urgent (when blood is needed in a day or two, and 3) Non-urgent (when blood is needed in about a week or more)

a. How would you describe the common situations in which your patients need blood: are they emergencies, urgencies or non-urgent?

    Note: If more than one pathway is chosen ask the next set of questions for each pathway

b. Can you please describe a scenario in the past when patients needed a blood transfusion as an <emergency/urgency/non-urgent>?
    - Prompt: What happened to these patients, and what were the outcomes? Did they get referred, or manage to get blood or something worse?

c. In a given month, how often might you request blood for similar patients?
d. How long does it take to get blood for these patients?
    - Prompt - how many hours, days, or weeks

e. How do shortages or delays in availability of blood affect these patients?
    - Prompt: Any outcomes, including delays, social, financial, clinical

f. What clinical criteria do you use to request blood in these patients?
    - Prompt: e.g. Hb or blood pressure or symptoms

g. What other (non-clinical) criteria do you use to request blood in these patients?
    - Prompt: e.g. social status, administrative or regulatory policies

h. What blood products do you commonly order for these patients?
i. Is there a greater need for blood products besides whole blood?

3. Estimating need for blood

a. Currently approximately how many units do you order in a month?
    - Prompt:Would you say it was: (<5, 5-10, 10-15, 15-20, >20 units)

b. Of these, how many units do you actually receive?
c. About how many times in a month have you received the blood you ordered, but not used all of it?
d. Prompt: Why does this happen?
e. In an ideal world, how many units of blood would you order in a month?

4. Challenges, barriers, and possible solutions

a. What, in your opinion, are the barriers to providing every patient needing blood with a blood transfusion?
    - Probe: Issues with donation/availability or processing or delivery

b. What solutions do you see to overcome these barriers??
    - Probe : Improvements in donation/availability or processing or delivery

c. Can you speak to/highlight any positive changes or negative changes you have noticed in the blood transfusion system in Kenya during your career?

d. Out of curiosity, have you ever donated blood?

Tool 2d: Individual Interviews with Patients and Caregivers

Introductory Prompt

Thank you for agreeing to meet with us. We are interested in understanding the blood need in your region/hospital and understanding how blood availability, processing, and delivery works. Through interviews we will be gaining perspective on current barriers and possible solutions.

Questions

1. Can you tell us the last time you received a blood transfusion?
2. Can you describe why you needed blood? What was your medical problem?
    a. Probe: How quickly was blood needed, and how much?
3. Can you describe what happened after you were told that you needed blood?
    a. Clarification: Did you or your family have to do anything once you knew that blood was needed?
4. Did you receive the blood you needed?
    a. Probe: Did you receive it in time? How long did it take from when you knew you needed blood to when you received blood?
    b. Probe: Do you think you received the amount of blood you needed?
5. Where were you when you received the blood ?
6. Did you face any challenges to receive the blood that you needed?
7. Have you ever donated blood?
    a. If yes: Can you tell us about when and why you donated?
    b. If no: Why not? What would make you donate blood?
8. Do you, or would you encourage your friends and family to donate blood?
    a. Probe: Why (not)?
9. What do you think stops people in your community from donating blood?
10. Given your experiences and challenges in receiving blood, what do you think could be done to improve the availability of blood for other patients like you?

Tool 3a: Focus Group Discussion (FGD) with Donors

Introductory Prompt

Thank you for agreeing to meet with us. We are interested in understanding the blood need in your region/health facility and understanding how blood availability, processing, and delivery works. Through interviews we will be gaining perspective on current barriers and possible solutions.

<u>FGD Questions</u>

1. **Blood Donation History**
    a. Approximately how many times have you ever donated blood in your lifetime?
    b. How many times have you donated blood in the last year?
    c. When did you last donate blood?
    d. Where have you donated the blood?
        i. PROBE: If you know the site, was it a National / Regional/Satellite Blood Transfusion Centre, or a/ Private Hospital, or a/ Public hospital, or in a /Blood Donation Drive in a school, church or other site)

2. **Blood Donation Experience**
    a. Can you describe a specific instance when you donated blood?
    b. Can you describe your experience so far with donating blood, either one experience or in general?
    c. Based on your experience(s), are you likely to donate blood again?
        i. Probe: Can you say why?

3. **Motivation for blood donation**
    a. Can you describe the reasons why you donated blood, either in one instance, or in general?
    b. Were you ever paid for giving blood, or compensated in other ways?
    c. What would motivate you to donate blood again?

4. **Mobilisation for blood donation**
    a. Do you think it is easy for people in your communities - your friends and relatives - to go and donate blood?
        i. Probe: What are the possible reasons that stop them from donating blood?

b. What in your mind would help more people in your communities to volunteer to donate blood?
c. Did you receive messages requesting you to donate blood, either from friends or the community or the health services?
    i. Probe: How did you get these messages and what effect did they have? Was it through word of mouth, radio, social media or in other ways?
d. Have there been requests for blood donation that you have not responded to?
    i. Probe: Can you say more about these requests and why you didn't respond?
e. How do you think people should be appreciated when they donate blood?
    i. Probe: For example, with some soda or snack, a certificate, or some other ways?

5. Challenges & potential solutions

a. What do you think the hospital, the county, or national government (or anyone else) could do to improve blood donation?
    i. Probe: What do you think can be done to encourage more volunteer blood donation (not only when a family member or friend needs blood)?
b. Would you encourage your friends and family members to voluntarily donate blood?
    i. Probe: Why (not)?

Tool 3b: Focus Group for the Medical and Clinical Officers (Cross-Cutting)

Introductory Prompt

Thank you for agreeing to meet with us. We are interested in understanding the blood need in your region/hospital and understanding how blood availability, processing, and delivery works. Through interviews and focus group discussions we will be gaining perspective on current barriers and possible solutions.

1. **Blood Logistics**
    a. **Role in blood transfusion process**
        i. Can you describe how you obtain blood for your patients?
            - Prompt: Can you describe the most recent clinical scenario where one of your patients needed blood, and what happened?
        ii. What are the most common conditions for which you order blood in your facility?
    b. **Blood process**
        i. Can you describe the steps for an MO/CO to put an order in for blood?
        ii. How might these steps change in a facility with a blood bank versus one without a blood bank, based on your experience in other facilities?
        iii. How might the steps change when ordering blood in outpatient versus inpatient settings?

2. **Clinical Pathways for blood transfusion**

    We would like to understand how blood is available depending on the urgency with which blood is needed: 1) Emergencies (e.g. trauma or PPH), 2) Urgent conditions (e.g. severe anaemia in children or adults), and 3) Non-urgent conditions (e.g. before chemotherapy or cancer surgery)

    a. Focussing now on emergencies (e.g. trauma or PPH)
        i. Do processes for ordering blood change if blood is needed in an emergency?
            1. If so, how?
        ii. Do you receive enough blood, and in time, to manage emergencies?
        iii. What do you do in these cases if enough blood is not received in time?
        iv. What additional steps do you take in case blood is needed in emergencies?
    b. Urgent conditions (e.g. severe anaemia in children or adults)
        i. Do processes for ordering blood change if blood is needed in urgent conditions?
            1. If so, how?
        ii. Do you receive enough blood, and in time, to manage urgent conditions?
        iii. What do you do in these cases if enough blood is not received in time?

iv. What additional steps do you take in case blood is needed in urgent conditions?

c. Non-urgent conditions (e.g. before chemotherapy or cancer surgery)

i. Do processes for ordering blood change if blood is needed in non-urgent conditions?

1. If so, how?

ii. Do you receive enough blood, and in time, to manage non-urgent conditions?

iii. What do you do in these cases if enough blood is not received in time?

iv. What additional steps do you take in case blood is needed in non-urgent conditions?

3. Estimating need for blood

a) Currently, approximately how many units do you order in a month? Prompt: Would you say it was (<5, 5-10, 10-15, 15-20, >20 units)

b) Of these, how many units do you actually receive?

c) About how many times in a month have you received the blood you ordered, but not used all of it? Prompt: Why does this happen?

d) In an ideal world, how many units of blood would you order in a month?

4. **Questions on blood products**

a) What blood products do you commonly order for these patients?

b) Is there a greater need for blood products besides whole blood?

5. Blood prioritisation

i. What are the commonest types of patients you order blood for?

ii. How do you prioritise patients for receiving blood?

iii. Who prioritises blood orders?

- Prompt: How does the system work if blood needs to be prioritised for certain patients?

iv. Do you sometimes decide to give blood to some patients over others? If so, can you tell us how and why you prioritise giving blood to one patient over another?

- Prompt: Do you sometimes give some patients less blood than they need because other needs are more important, or delay giving blood to some patients until other patients get blood

v. What happens when there is a stockout/shortage?

- Prompt : What do you do, or what steps do you take?

6. Challenges, barriers, and possible solutions

a) What, in your opinion, are the barriers to providing every patient needing blood with a blood transfusion?

- ■ Probe: Issues with donation/availability or processing or delivery

b) What solutions do you see to overcome these barriers??

- ■ Probe : Improvements in donation/availability or processing or delivery

c) Can you speak to/highlight any positive changes or negative changes you have noticed in the blood transfusion system in Kenya during your career?

- ○ Out of curiosity, have you ever donated blood?

Tool 3c: Focus Group Discussion with Inpatient Nurse/Midwives

Introductory Prompt

Thank you for agreeing to meet with us. We are interested in understanding the blood need in your region/hospital and understanding how blood availability, processing, and delivery works. Through interviews we will be gaining perspective on current barriers and possible solutions.

1. **Blood Logistics**
   a. **Role in blood transfusion process**
      i. Can you describe your role in obtaining blood for your patients
         - Prompt: Can you describe the most recent clinical scenario where one of your patients needed blood, and what happened?

      ii. What are the most common conditions for which you administer blood in your facility?

   b. **Blood process**

      i.How does a clinician put an order in for blood?

      - Prompt: Can you describe the steps?
      - Do you follow a specific protocol for administering blood or blood products?

      ii. How might these steps change in a facility with a blood bank versus one without a blood bank, based on your experience in other facilities?

      iii.If you work (or have worked in different hospitals), have you seen differences in how you order and receive blood at those hospitals?

2. **Clinical pathways for blood transfusion**

   We would like to better understand your practice around blood transfusion for the following three clinical situations: 1) Emergencies (when blood is needed in a matter of hours), 2) Urgent (when blood is needed in a day or two, and 3) Non-urgent (when blood is needed in about a week or more)

   - How would you describe the common situations in which your patients need blood: are they emergencies, urgencies or non-urgent?

   - Focussing now on emergencies (e.g. trauma or PPH)
     - Do processes for accessing and transfusing blood change if blood is needed in an emergency?
       - If so, how?
     - Do you receive enough blood, and in time, to manage emergencies?

    - What do you do in these cases if enough blood is not received in time?
    - What additional steps do you take in case blood is needed in emergencies?
    - Probe: what other blood substitutes are used?
- Urgent conditions (e.g. severe anaemia in children or adults)
    - Do processes for accessing and transfusing blood change if blood is needed in urgent conditions?
        - If so, how?
    - Do you receive enough blood, and in time, to manage urgent conditions?
    - What do you do in these cases if enough blood is not received in time?
    - What additional steps do you take in case blood is needed in urgent conditions?
- Non-urgent conditions (e.g. before chemotherapy or cancer surgery)
    - Do processes for accessing and transfusing blood change if blood is needed in non-urgent conditions?
        - If so, how?
    - Do you receive enough blood, and in time, to manage non-urgent conditions?
    - What do you do in these cases if enough blood is not received in time?
    - What additional steps do you take in case blood is needed in non-urgent conditions?

3. Blood Prioritisation

i. In the process of administering ordered blood to patients. If a scenario occurs where two patients have the same blood group, how do you decide who to transfuse and who not to transfuse?

- Prompts: What happens in such a scenario? Do they get delayed or do they miss the blood entirely?

ii.Can you describe a scenario where a patient needed blood and they did not get it? What happened?

    - Probe: How often do you miss blood for patients who need transfusion? Do you miss most of the time or sometimes? Do you give some patients less blood than they need because other needs are more important, or delay giving blood to some patients until other patients get blood?

iii.What are the commonest types of patients you transfuse blood for?

iv.What happens when there is no blood in the hospital?

- Prompt : What do you do, or what steps do you take?

4. **Estimating need for blood**

a) Currently approximately how many units is ordered in a month in your ward?

Prompt: Would you say it was (<5, 5-10, 10-15, 15-20, >20 units)

b) Of these, how many units do you actually receive?

c) About how many times in a month have you received the blood you ordered, but not used all of it?

Prompt: Why does this happen?

d) In an ideal world, how many units of blood would be ordered in a month in your ward?

5. **Challenges and possible solutions to blood transfusion**

i.Can you describe the types of patients in need of blood that you deal with on a daily basis?
ii.Do you encounter any challenges in administering blood to patients?

- PROBE: What are the most common challenges?

iii.What do you see as the main barrier to every patient in need of transfusion receiving the necessary blood transfusion?

iv.What solutions do you see to overcome these barriers??

- Probe : Improvements in donation/availability or processing or delivery
- Probe : Is there anything you have done in the past to help your patient receive the blood they need?

v. Can you speak to/highlight any positive changes or negative changes you have noticed in the blood transfusion system in Kenya during your career?

vi.Out of curiosity, have you ever donated blood?
